# Epidemiological Trends and Clinical Profile of Dengue in Assam: A Retrospective Analysis From 2022 to 2025

**DOI:** 10.7759/cureus.102801

**Published:** 2026-02-01

**Authors:** Hiramoni Sarmah, Sanjay Bhattacharya, Derhasar Brahma, Sristi Majumdar, Jahnabi Gogoi, Elmy S Rasul

**Affiliations:** 1 Microbiology, Virus Research Diagnostic Laboratory, Fakhruddin Ali Ahmed Medical College and Hospital, Barpeta, IND; 2 Microbiology, Fakhruddin Ali Ahmed Medical College and Hospital, Barpeta, IND; 3 Veterinary Microbiology, Virus Research Diagnostic Laboratory, Fakhruddin Ali Ahmed Medical College and Hospital, Barpeta, IND; 4 Medical Microbiology, Fakhruddin Ali Ahmed Medical College and Hospital, Barpeta, IND

**Keywords:** clinical manifestation, dengue virus, elisa, epidemiology, retrospective study

## Abstract

Background

Dengue causes a significant public health challenge in northeastern India, yet current epidemiological data from Assam remain limited. This study examines the epidemiological profile, demographic characteristics, seasonal trends, and clinical presentation of dengue cases reported from a tertiary care hospital in Barpeta district, Assam.

Methods

This hospital-based retrospective study was conducted from 2022 to 2025. A total of 1,119 clinically suspected dengue patients were screened for dengue virus-specific NS1 antigen and immunoglobulin M (IgM) antibodies using IgM antibody capture enzyme-linked immunosorbent assay (MAC-ELISA). Demographic details, seasonal variation, and clinical manifestation of laboratory-confirmed cases were analyzed.

Results

A total of 1,119 clinically suspected cases were considered, out of which 152 (13.6%) were laboratory-confirmed for dengue infection. Dengue positivity exhibited noticeable year-to-year variability, with the highest burden recorded in 2023. Males were incommensurately affected, and the 21-40-year age group constituted the largest share of confirmed cases, indicating increased transmission among the physically active population. A striking seasonal pattern was observed, characterized by a substantial rise in cases during the monsoon and post-monsoon periods, consistent with vector proliferation during periods of high rainfall and humidity. Urban residents were associated with a higher incidence of dengue infection compared to rural settings. The most widely reported clinical manifestations included fever, headache, and musculoskeletal pain.

Conclusion

Overall, the findings emphasize that the sustained burden of dengue in the regionand underscore the importance of strengthening vector monitoring systems, executing timely preventive measures, and enhancing early diagnostic capacity to take the edge of future outbreaks.

## Introduction

Dengue fever is an acute systemic mosquito-borne viral disease worldwide, posing a substantial public health burden in tropical and subtropical regions. The disease is caused by five antigenically distinct serotypes of Dengue Virus 1 to 5 (DENV-1 to DENV-5), belonging to the genus *Flavivirus* of the family Flaviviridae [1,2]. Transmission occurs primarily through the bites of infected Aedes aegypti and Aedes albopictus mosquitoes, which thrive in densely populated urban and peri-urban environments. Global incidence of dengue is evidently increasing over the past 5 decades, with an estimated 390 million infections occurring annually, of which nearly 96 million manifest clinically [3].

In India, dengue has evolved from an epidemic infection to an endemic disease with hyper-endemic circulation in several states. Climatic factors, such as temperature, humidity, and rainfall, combined with rapid urbanization, water storage practices, and inadequate vector control measures, contribute significantly to its spread [4]. The northeastern region of India has historically reported lower dengue transmission compared to other parts of the country [5]. However, in the past decade, several states, including Assam, Arunachal Pradesh, and Manipur, have experienced sharp rises in incidence and periodic outbreaks [6,7]. Assam, in particular, presents an emerging hotspot with sporadic outbreaks since the early 2000s and a notable increase in transmission intensity in recent years [8].

Timely diagnosis plays a critical role in reducing dengue-related morbidity and mortality. Early identification of cases enables appropriate clinical management and facilitates public health responses. The National Vector Borne Disease Control Programme (NVBDCP) recommends the use of NS1 antigen detection during the early febrile phase and IgM by IgM antibody capture enzyme-linked immunosorbent assay (MAC-ELISA) for serological confirmation from day five of illness onward. These diagnostic tools remain essential in resource-limited settings where molecular assays may not be routinely available. Despite expanding diagnostic facilities, gaps persist in understanding the epidemiology of dengue across many districts in Assam, where many factors, like underreporting and limited surveillance capabilities, hinder accurate assessments.

Considering these gaps, the present study was undertaken for a detailed four-year retrospective analysis of dengue cases diagnosed at Fakharuddin Ali Ahmed Medical College and Hospital (FAAMCH), Barpeta, a major tertiary care hospital in Assam. The study examines temporal patterns, demographic characteristics, seasonal fluctuations, geographic distribution, and clinical manifestations of laboratory-confirmed dengue cases from 2022 to 2025. This study will help in strengthening local vector surveillance strategies, improving the timing and targeting of control interventions, and supporting data-driven public health planning. By providing updated epidemiological insights, this work contributes to the growing body of literature on dengue in northeastern India and highlights the continued need for integrated surveillance and early diagnosis to mitigate the disease burden.

## Materials and methods

This retrospective observational study was carried out in the virus research diagnostic laboratory (VRDL) of FAAMCH, a tertiary care teaching hospital located at Barpeta, Assam, India. The hospital serves as a major diagnostic and referral centre for neighbouring districts and caters to a diverse population spanning both rural and urban settings. All dengue-related diagnostic investigations conducted over four years, from January 2022 to December 2025, were reviewed. The study population consisted of 996 outpatients and 123 inpatients who underwent laboratory testing for dengue based on clinical suspicion. Clinicians identified suspected dengue cases following the WHO guidelines. This work was presented to and approved by the institutional ethical committee of FAAMCH (FAAMCH/Ethics Committee/128/Pt./2022/1842). Suspected cases included individuals presenting with acute febrile illness, accompanied by headache, myalgia, arthralgia, retro-orbital pain, nausea, vomiting, or rash. Patients exhibiting other signs (e.g., abdominal pain, mucosal bleeding, lethargy, hepatomegaly) were also considered as suspected. No exclusion was applied for age, sex, pregnancy status, comorbid conditions, or prior history of dengue. All consecutive cases for whom dengue diagnostic tests were requested were considered eligible. Patients with inadequate blood volume, hemolyzed samples, or missing laboratory numbers were excluded from laboratory testing but noted in the suspected case count when traceable. Aseptically collected venous blood samples, labeled with a unique number, were sent to the VRDL within 30-60 minutes of collection. Serum was separated by centrifugation at 3000 rpm for 10 minutes and processed immediately to maintain antigen and antibody integrity. In cases requiring delayed testing, serum aliquots were stored at 2-8 °C for a maximum of 24 hours.

Patients presenting within the first five days of fever onset were tested for dengue-specific NS1 antigen, using commercially available ELISA kits. The tests were conducted according to the manufacturer’s recommendations. Quality control was ensured through the daily verification of kit performance using internal controls. Positive NS1 results were considered confirmatory for acute dengue infection. Serological detection of anti-dengue IgM antibodies was performed using standardized IgM MAC-ELISA kits for patients presenting after day five of illness or when NS1 antigen results were negative despite clinical suspicion. The entire ELISA protocol involving the preparation and equilibration of reagents, dilution of serum samples, incubation with anti-human IgM capture antibodies, dengue antigen conjugate, and chromogenic substrate, with recommended washing cycles, was followed as per the instructions of the ELISA kit. Measurement of optical density (OD) values using a calibrated automated microplate ELISA reader. Each assay included positive control, negative control, and calibrator wells. Borderline results were repeated before final interpretation.

Demographic information like age, sex, residential district, and rural-urban status was collected from laboratory requisition forms, while the clinical features and presenting symptoms were extracted from the case record form. Collected data related to the monthly and yearly distribution of dengue cases were cross-checked to ensure their completeness and consistency. Mortality data were included only when dengue positivity was laboratory-confirmed and the treating clinician certified dengue as a direct or contributory cause of death. Graphical representations were generated to illustrate seasonal patterns and demographic distributions. Patterns and trends were compared across years to identify fluctuations in dengue positivity. Confidentiality and data security were rigorously maintained throughout the research process.

## Results

A total of 210 fever cases were reported in 2022, which sharply increased to 430 cases in 2023. In 2024 and 2025, reported clinical cases declined to 246 and 233, respectively. Laboratory confirmation was done by ELISA, with the year-wise highest positivity in 2024 (40, 16.2 %) and the lowest in 2025 (17, 7.3%). Overall, a total of 1,119 suspected cases and 152 confirmed cases were recorded during this timeframe, with a dengue prevalence of 13.6%. The year-wise distribution of suspected and confirmed cases is presented in Table 1.

**Table 1:** Year-wise distribution of dengue cases

Year	No. of clinical cases (Suspected)	Dengue-positive cases (Confirmed)		
		NS1 Antigen	IgM	Total (%)
2022	210	8	24	32 (15.2%)
2023	430	7	56	63 (14.6%)
2024	246	4	36	40 (16.2%)
2025	233	3	14	17 (7.3%)
Total (In four Years)	1119	22	130	152 (13.6%)

The 152 laboratory-confirmed dengue cases were analyzed for demographic characteristics. The 21-40-year age group constituted the highest proportion of dengue cases (68, 44.7%), followed by the 41-60-year age group contributing 39 cases (25.7%), both representing high-risk occupationally active groups. The 0-20-year group accounted for 28 cases (18.4%), while all the suspects above the age of 61 accounted for 17 cases (11.2%). Gender distribution revealed a clear male predominance, with 101 males (66.4%) and 51 females (33.6%) testing positive. Age-group and gender distributions are summarized in Table 2.

**Table 2:** Distribution of dengue cases by age group and gender

Age group in years	Males (%, n=152)	Females (%, n=152)	Total cases (%, n=152)
0-20	17 (11.2%)	11 (7.2%)	28 (18.4%)
21-40	49 (32.2%)	19 (12.5%)	68 (44.7%)
41-60	27 (17.7%)	12 (7.9%)	39 (25.6%)
>61	8 (5.2%)	9 (5.9%)	17 (11.2%)
Total	101 (66.4%)	51 (33.5%)	152 (100%)

**Figure 1:**
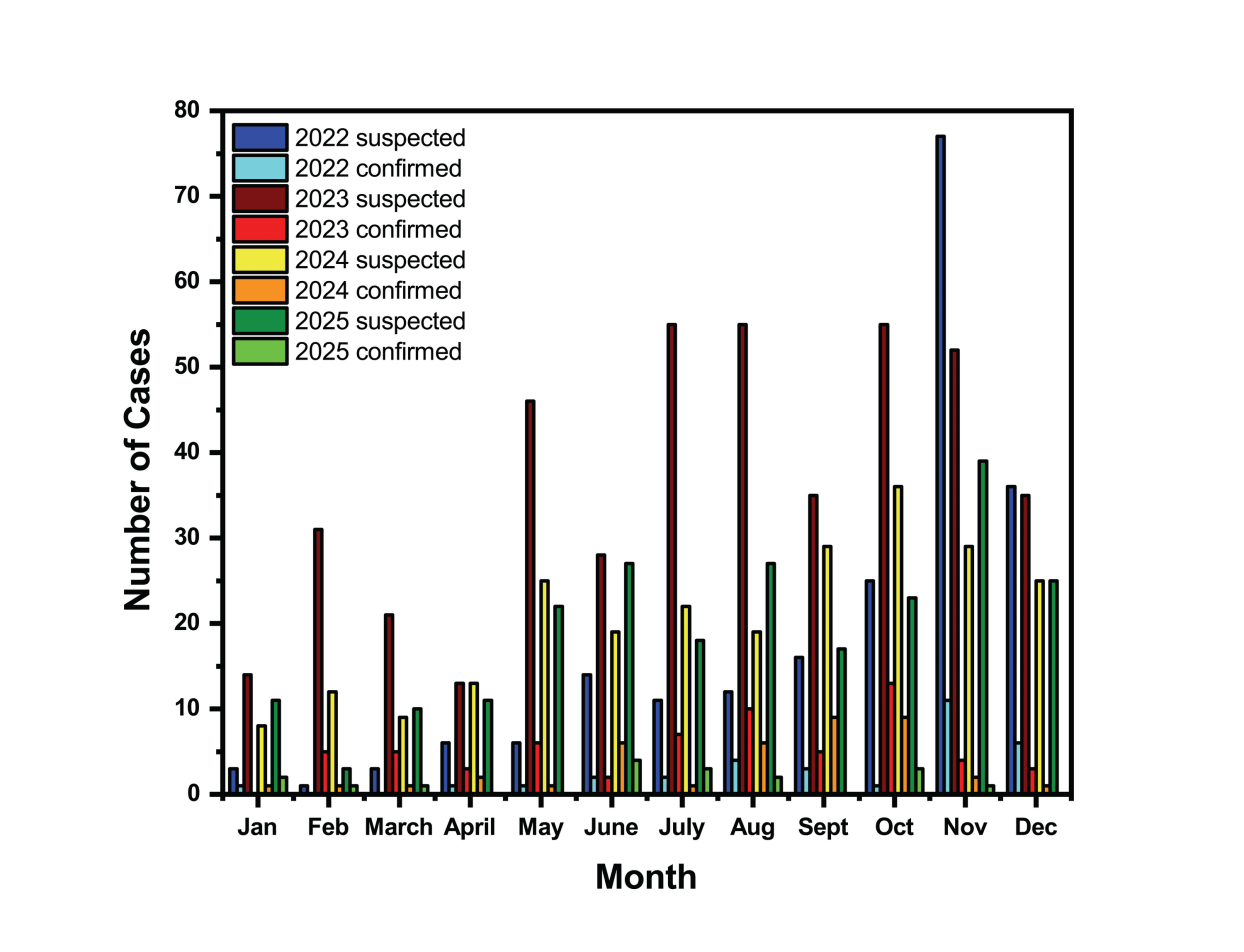
Month-wise distribution of dengue cases from 2022 to 2025

Figure 1 represents the monthly distribution of suspected and confirmed dengue cases over the four-year study period from 2022 to 2025. The year 2023 exhibited the highest burden of both suspected (n=430) and confirmed (n=63) cases, representing approximately 38% and 42% of the cumulative totals (1119 and 152), respectively. In contrast, 2022 had the lowest number of suspected cases (n=210), while 2025 recorded the fewest confirmed cases (n=17). More suspected cases were detected during the latter months, particularly from September to December. For instance, November consistently showed elevated suspected cases in most years (ranging from 29 in 2024 to 77 in 2022), and confirmed cases peaked in November 2022 (n=11) and October 2023 (n=13). Across all four years, dengue detection was lowest from January to April, with suspected cases rarely exceeding 20 per month and confirmed cases often at or near zero.

Dengue prevalence differed significantly across geographical settings. Out of the total 152 confirmed cases, urban areas contributed 93 cases (61.18%) and rural areas contributed 59 cases (38.82%). The rural-urban distributions are given in Table 3.

**Table 3:** Distribution of dengue cases in rural and urban areas

Area	Total number of cases	Percent cases
Urban	93	61.18
Rural	59	38.82

Clinical manifestations among the 152 confirmed dengue cases reveal that fever was universal (100%), consistent with the characteristic of dengue infection. Other predominant symptoms included headache (70.39%), arthralgia/myalgia (51.97%), nausea/vomiting (40.13%), and retro-orbital pain (28.29%). These findings are characteristic of classical dengue fever. Severe or warning signs were comparatively less frequent: petechiae (2.63%), seizures (1.97%), unconsciousness (0.66%), and altered mental status (0.66%). This distribution is outlined in Table 4.

**Table 4:** Clinical presentation of dengue-positive cases

Clinical Presentation	Number	Percentage
Fever	152	100
Headache	107	70.39
Arthralgia and myalgia	79	51.97
Nausea and vomiting	61	40.13
Retro-orbital pain	43	28.29
Petechiae	4	2.63
Seizures	3	1.97
Unconsciousness	1	0.66
Altered mental status	1	0.66

## Discussion

The present study provides an extensive epidemiological and clinical overview of dengue infection in the region over four consecutive years (2022-2025), revealing critical insights into transmission dynamics, demographic vulnerability, clinical patterns, and geographical clustering. Over the study period, dengue infection was confirmed in 13.6% of suspected cases, indicating ongoing transmission in this region of Assam. Similar studies on dengue from Assam and other parts of northeastern India have reported dengue positivity rates ranging from 10% to 25%, supporting the endemicity [9,10]. The rise in suspected dengue cases in 2023, followed by a decline in subsequent years, suggests inter-annual variability in dengue transmission, possibly influenced by climatic factors, vector density, and healthcare-seeking behavior [11].

The 21-40 year age group constituted the highest proportion (44.7%) of infections, likely due to increased outdoor exposure, occupational mobility, and greater interaction with mosquito habitats. The 41-60-year age group represented another high-risk, occupationally active group (25.7%). Gender distribution revealed a clear male predominance, with 66.4%, while 33.6% of females tested positive. This gender-based variation might be due to exposure differences, behavioral patterns, and outdoor activity levels that facilitate Aedes aegypti bites. This finding is consistent with observations from multiple Indian studies, where greater outdoor exposure and occupational mobility have been associated with higher dengue risk [10,12]. The observed male predominance further reflects differential exposure patterns and healthcare utilization, a trend widely reported in dengue epidemiology across India [13].

The monthly distribution of dengue cases across four years demonstrated a distinct seasonal pattern, strongly associated with monsoon and post-monsoon climatic conditions. The lowest incidence of dengue cases was observed during the months of January to April, reflecting an unfavorable breeding season for mosquitoes, resulting in low viral transmission. Considering the pre-monsoon period, starting from May to July, which promotes mosquito breeding, with a peak in November. A similar trend of dengue incidence was observed throughout the study period, with a gradual decrease in the number of cases since 2024 onward. Comparable seasonal trends have been documented in other districts of Assam, emphasizing the predictable post-monsoon surge of dengue in the state [9,14,15].

The higher urban burden aligns with known epidemiological patterns, where Aedes aegypti thrives in densely populated environments with artificial water collections like construction sites, discarded containers, and poorly managed drainage systems [16-18]. In contrast, rural areas reported comparatively a less number of cases, likely due to lower population density and different water storage behaviors. Previous studies like Bowman et al. (2016) and Kraemer et al. (2015) also suggested that rapid urbanization, higher population density in urban settings, and inadequate solid waste management help produce high vector densities and thereby increase vector and human contact, which facilitates a higher burden of dengue in urban areas [19,20].

Clinical manifestations among the 152 confirmed dengue cases reveal that fever was universal (100%) and may be considered characteristic of dengue infection. Other predominant symptoms included headache (70.39%), arthralgia/myalgia (51.97%), nausea/vomiting (40.13%), and retro-orbital pain (28.29%). These findings are characteristic of classical dengue fever. Severe or warning signs were comparatively less frequent with petechiae (2.63%), seizures (1.97%), unconsciousness and altered mental status (0.66%), each. Similarly, in a study by Gobler et al. (2016), fever was observed in all cases [21], and it plays its role as the most consistent and defining clinical feature of dengue infection. Few studies show that the detection of neurological manifestations like seizures and altered mental status, although rare, is clinically significant [22].

As the study is retrospective and hospital-based, the findings are likely not representative of the whole community or the districts related to the hospital. Asymptomatic or mildly symptomatic dengue cases that did not visit the hospital were left underreported. As molecular confirmation and serotyping of the dengue virus were not performed, circulating serotypes in this particular case could not be known, which is to be done in due course of time. A detailed study on vector population, its breeding pockets, etc., has to be done with more accurate entomological and environmental data. Despite the above-mentioned limitations, the study provides valuable information about the epidemiological trend, seasonal variation, and clinical manifestations of dengue in Barpeta and its nearby districts.

## Conclusions

The overall findings of this study strengthen key public health priorities like enforcing early-warning systems based on climatic and entomological indicators, implementing district-specific vector control, enhancing diagnostic capabilities, including combined NS1-IgM testing, and waste management. In conclusion, the findings of this study reinforce the need for sustained, climate-linked, and region-specific dengue control strategies, guided by local epidemiological data and supported by national and international health frameworks.
